# Advances in the Molecular Mechanisms of Resistance in *Chilo suppressalis*

**DOI:** 10.3390/insects16090942

**Published:** 2025-09-08

**Authors:** Wenchao Ge, Guanghang Chen, Mengzhen Wang, Shunfan Wu, Congfen Gao

**Affiliations:** State Key Laboratory of Agricultural and Forestry Biosecurity, State & Local Joint Engineering Research Center of Green Pesticide Invention and Application, College of Plant Protection, Nanjing Agricultural University, Nanjing 210095, China; gewenchao95@163.com (W.G.); 13868522586@163.com (G.C.); 13965261983@163.com (M.W.); wusf@njau.edu.cn (S.W.)

**Keywords:** *Chilo suppressalis*, insecticide resistance, resistance mechanism, target- insensitivity, metabolic detoxification

## Abstract

The rice stem borer, *Chilo suppressalis* (Walker) (Lepidoptera: Crambidae), is one of the major pests in rice-growing areas. Its larvae feed on rice stems, causing symptoms of rice dead sheaths, dead hearts, and withered ears, resulting in heavy rice yield losses. Chemical insecticides remain the cornerstone of control strategies; however, the rapid development of resistance to multiple insecticide classes has emerged as a critical challenge to farmers and pest control specialists. Advanced methods utilizing molecular and gene sequence data from field-collected *C. suppressalis* populations, both resistant and susceptible, have provided a deeper understanding of the resistance mechanisms in this pest. Several components of Insecticide Resistance Management (IRM) programs serve as countermeasures to insecticide resistance in this pest. In the current review, we concentrate on insecticide resistance development, molecular mechanisms and resistance management of *C. suppressalis*.

## Simple Summary

The rice stem borer is a devastating pest in paddy fields, inflicting considerable damage across all growth stages of the rice. In China, the economic losses attributed to this pest are estimated at approximately CNY 11.5 billion annually. To safeguard rice production, pest management strategies have predominantly relied on the application of chemical insecticides. In recent years, the emergence and escalation of insecticide resistance in *Chilo suppressalis* have posed a growing threat to food security. This review provides a comprehensive overview of the resistance evolution of *C. suppressalis* to various classes of insecticides, including avermectins, organophosphates, macrolides, diamides, and diacylhydrazines. Particular emphasis is placed on elucidating the molecular mechanisms underlying resistance, with a focus on both target-site insensitivity and metabolic detoxification. Collectively, this work aims to offer a theoretical foundation and practical guidance for the sustainable management of insecticide resistance in *C. suppressalis*.

## 1. Introduction: The Occurrence and Damage of *Chilo suppressalis*

*Chilo suppressalis* (Walker) (Lepidoptera: Crambidae) is a devastating rice pest that is distributed extensively across global rice-producing regions and has established itself as a persistent threat to rice production systems [1]. In China, *C. suppressalis* is prevalent in nearly all rice-growing provinces, with particularly severe outbreaks recorded in the middle and lower reaches of the Yangtze River, including Zhejiang, Jiangxi, Hunan, Hubei, Sichuan, northern Jiangsu, Shaanxi, Henan, and Yunnan [2]. The spatiotemporal dynamics of *C. suppressalis* are strongly influenced by shifts in agricultural practices. During the 1950s, under a prevalent system of single-season or rotational rice cultivation, *C. suppressalis* was the dominant pest species. However, its population declined during the 1960s–1970s following the widespread implementation of double-cropping rice systems. In contrast, the rapid expansion of hybrid rice after the 1980s led to a resurgence in population densities, culminating in frequent large-scale outbreaks by the mid-1990s. Concurrently, rising temperatures associated with climate change have extended the pest’s active season and increased the number of annual generations, further intensifying its impact on rice production [3,4,5].

The number of generations per year of *C. suppressalis* displays marked geographic variation, influenced by latitude, climatic conditions, and regional cropping structures. It typically completes one to five generations annually, with a general increase in generational frequency from north to south [6]. In northeastern China, one to two generations are common [7]; in the Jianghuai region, two generations prevail; in southwestern rice areas, two to four generations may occur; and in the middle and lower Yangtze River basin, four complete generations are now typical, with the second and third generations often causing the most serious yield losses [8,9]. Beyond climatic and geographic factors, the type of host plant also exerts a significant influence on the biology of *C. suppressalis*. Notably, when feeding on *Zizania latifolia* (water bamboo), *C. suppressalis* exhibits a faster development rate, enhanced pupal weight, improved uniformity, and higher reproductive capacity [10].

*C. suppressalis*, as a polyphagous insect, has a host range that extends far beyond rice, also widely infecting crops such as *Z. latifolia*, sorghum, corn, wheat, barnyardgrass, broad beans, and rapeseed, reflecting its high adaptability and broad damage potential [11]. The insect inflicts damage across all growth stages of the rice plant. Mature larvae of the overwintering generation remain in rice stubble, pupating in spring as temperatures rise. After adult emergence, oviposition occurs preferentially on broader leaves of robust rice tillers. Neonates initially feed on leaf sheaths, producing chlorotic lesions, and subsequently tunnel into the culm, disrupting the plant’s vascular system. During the tillering stage, infestation results in sheath blight and dead heart symptoms; at panicle initiation, damage manifests as panicle degeneration; at heading, as withered ear formation; and during the grain-filling stage, as half-filled panicles and injured culms [12,13].

## 2. Advances in Insecticide Resistance in *C. suppressalis*

Chemical control has long been the cornerstone of field suppression efforts, owing to its rapid efficacy, operational efficiency, cost-effectiveness, and minimal constraints in terms of geography and seasonality. However, the sustained and often unregulated application of insecticides has precipitated a significant escalation in resistance development within *C. suppressalis* populations, thereby undermining the long-term effectiveness of chemical interventions and posing serious challenges to integrated pest management (IPM) programs [14].

As of 2025, a total of 28 single active ingredients have been registered in China for chemical control of *C. suppressalis*. Nonetheless, resistance has been documented to a substantial proportion of these compounds across multiple insecticide classes, including nereistoxins, organophosphates, macrocyclic lactones, and diamides [15,16]. The magnitude of resistance varies across geographical regions, a pattern that is closely associated with differences in pesticide application intensity, frequency, and historical usage practices. These trends collectively underscore the urgent need to elucidate resistance mechanisms and implement science-based resistance management strategies [17].

### 2.1. Evolution of Resistance to Nereistoxin Insecticides in C. suppressalis

Nereistoxin insecticides, such as monosultap, have historically played a pivotal role in the control of *C. suppressalis* due to their potent contact, stomach, and systemic activities. However, intensive and prolonged reliance on this single class of insecticides led to the widespread emergence of resistance and a marked reduction in field control efficiency [18].

The evolution of resistance followed a distinct trajectory. Moderate resistance to monosultap and bisultap was detected in major rice-growing provinces as early as 1986–1990 [19]. In 1999, field populations from several regions in Zhejiang province were first documented to exhibit high-level resistance to monosultap [20]. Subsequent resistance monitoring in 2004 revealed that *C. suppressalis* populations in Yongxin, Shangrao, and Fengcheng of Jiangxi province had similarly developed high-level resistance [21]. In light of increasing resistance pressure, the application of monosultap was gradually reduced. This shift in alternative insecticides led to a gradual decline in resistance levels since 2010. This pattern aligns with the “fitness cost” hypothesis, where resistant alleles are counter-selected in the absence of insecticide exposure. Subsequent monitoring from 2015 to 2019 confirmed this trend, revealing that resistance had largely regressed from 2.7 to 13.5-fold across most rice-growing regions [15,22].

### 2.2. Evolution of Resistance to Organophosphate Insecticides in C. suppressalis

Organophosphate (OP) insecticides were widely used to control *C. suppressalis* since the 1960s, while systematic resistance monitoring was carried out with the widespread adoption of triazophos in the 1990s; the rapid evolution of high to extremely high resistance was detected in Zhejiang province. Resistance ratios (RRs) exceeding 100-fold in many populations, and the Cangnan population reached 2367.3-fold in 2004 [23,24,25,26,27].

A moderate resistance (RR = 10.8–57.8-fold) of chlorpyrifos was detected in several provinces from 2006 to 2009 [27,28]. Nationwide surveys from 2015 to 2018 confirmed that resistance was widespread, with moderate to high levels persisting for triazophos (RR = 64.5–461.3-fold) and moderate to high levels developing for chlorpyrifos (RR = 10.1–125.0-fold) [22,29]. Recent surveillance from 2020 to 2022 in Hubei Province indicates that *C. suppressalis* populations continue to exhibit moderate resistance to both triazophos and chlorpyrifos, with resistance ratios ranging from 41.9 to 81.6-fold [30,31]. The long-term, intensive use of organophosphates has driven the evolution of significant resistance across China, underscoring the critical need for region-specific resistance management strategies based on continuous monitoring and judicious insecticide rotation.

### 2.3. Evolution of Resistance to Phenylpyrazole Insecticides in C. suppressalis

Phenylpyrazole insecticides, particularly fipronil, were introduced to China in the 1990s and quickly gained recognition for their strong field efficacy against a variety of agricultural pests, including *C. suppressalis.* Early studies reported low resistance in Jiangxi Province in 2004 [26], while subsequent monitoring from 2007 to 2009 revealed moderate resistance levels (RR = 11.2–27.0-fold) in Zhejiang Province. Notably, a *C. suppressalis* population from Jiangshan (Zhejiang) exhibited an increase in resistance from 6.8-fold in 2008 to 23.4-fold in 2009 [32]. Despite their effectiveness, fipronil was banned from application to paddy fields because of its high toxicity to non-target organisms such as bees and aquatic species like fish and shrimp in China since 1 October 2009.

### 2.4. Evolution of Resistance to Macrolide Insecticides in C. suppressalis

Macrolide insecticides, such as abamectin, emamectin benzoate, spinosad, and spinetoram, are currently used for the control of *C. suppressalis*. Resistance monitoring showed that field populations remained susceptible to abamectin before 2001, but developed moderate resistance in various provinces from 2004 [33,34]. since the mid-2010s, the resistance of some populations collected from Zhejiang and Hunan provinces has reached up to 94.1-fold [22,35], but remained sensitive to spinosad and spinetoram [29]. From 2020 to 2023, resistance of abamectin intensified significantly; several populations in Hunan and Hubei provinces developed high to extremely high resistance (RR = 123.5–443.5-fold) [31,36,37]. This progressive intensification of abamectin resistance highlights the growing threat to the control of abamectins on *C. suppressalis*.

### 2.5. Evolution of Resistance to Diacylhydrazine Insecticides in C. suppressalis

Diacylhydrazine insecticides such as tebufenozide, chromafenozide, and methoxyfenozide are employed for the control of *C. suppressalis*. Initial monitoring in 2016 revealed low to moderate resistance to methoxyfenozide in several field populations from Zhejiang, Hunan, and Henan provinces [38]. A subsequent comprehensive resistance survey from 2017 to 2023, however, demonstrated a significant and widespread escalation of resistance. By 2023, 50% of the populations exhibited high-level resistance (RR = 100.4–156.5-fold). High-level resistance was particularly prevalent in populations from Zhejiang and Jiangxi provinces and also noted in select populations from Hunan and Shanghai. Additionally, resistance levels in two Sichuan populations showed a marked increase compared to previous years. These results highlight a concerning trend of escalating methoxyfenozide resistance in *C. suppressalis* populations over both time and geographical space. Continued monitoring and integrated management strategies will be crucial for mitigating the further spread of resistance and maintaining the efficacy of diacylhydrazine-based insecticides in rice pest control [39].

### 2.6. Evolution of Resistance to Diamide Insecticides in C. suppressalis

**Figure 1:**
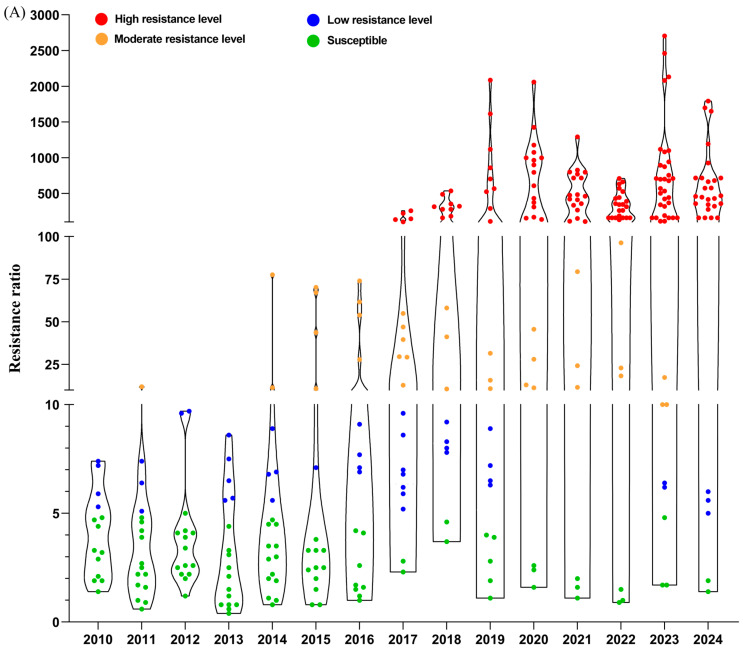

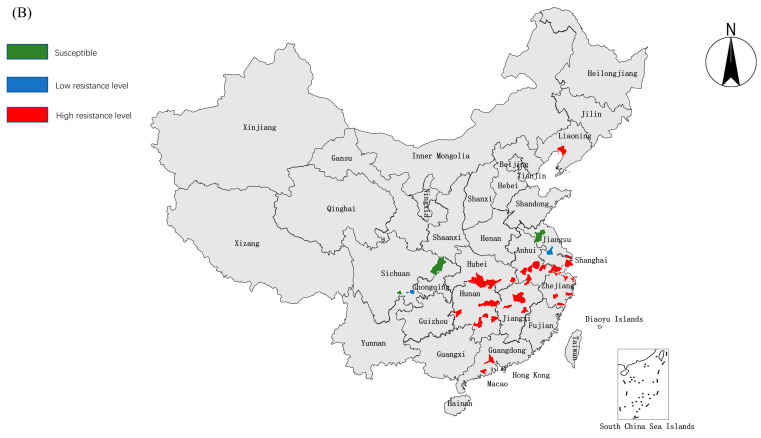
Dynamic monitoring of the chlorantraniliprole resistance status of *C. suppressalis* across China from 2010 to 2024 (**A**); Geographical distribution of *C. suppressalis* associated with chlorantraniliprole resistance in China in 2024 (**B**). Data were obtained from our previous study [16,44,46,47,48].

Diamide insecticides, particularly chlorantraniliprole, were introduced in 2008 and became a cornerstone for managing *C. suppressalis* due to their high efficacy and favorable safety profile. However, the extensive use of chlorantraniliprole led to the rapid development of resistance. Within a few years of its registration, resistance to chlorantraniliprole began to emerge in *C. suppressalis* populations (Figure 1A). Resistance monitoring from 2010 to 2011 in several regions of China, including Dong’an (Hunan), Rui’an, Longyou, and Xiangshan (Zhejiang), Huangshan and Lujiang (Anhui), and Yizheng (Jiangsu), revealed the onset of low-level resistance (RR = 5.1–7.4-fold) to chlorantraniliprole in *C. suppressalis* populations [40].

By 2012, most populations of *C. suppressalis* from various regions of Zhejiang, Hunan, Hubei, Jiangxi, and Anhui still showed sensitivity to chlorantraniliprole and flubendiamide, although some areas exhibited low to moderate levels of resistance [41,42,43]. In 2014, resistance monitoring revealed that the Yuyao population in Zhejiang had already developed moderate resistance to chlorantraniliprole (77.6-fold) [44]. By 2017, monitoring results indicated that populations from Yuyao (Zhejiang) and Cangnan (Zhejiang) exhibited high-level resistance (RR = 135.0–141.1-fold) [45]. Resistance levels continued to increase, with monitoring results from 2018 to 2019 showing a significant rise in resistance, particularly in populations from the Yangtze River basin, where the Xiangshan population exhibited a resistance ratio as high as 2087.5-fold in 2019 [46,47]. By 2021–2022, approximately 75% of monitored populations showed high-level resistance to chlorantraniliprole, and the number of resistant populations had markedly increased [16]. An evaluation of the sensitivity of 71 field populations of *C. suppressalis* to chlorantraniliprole in China during 2023–2024 revealed that the proportion of populations exhibiting high-level resistance increased to 80% (RR = 111.6–2706.4-fold) in 2023 and further rose to 90.3% (RR = 160–1794.7-fold) in 2024. The high resistance area extended from the Yangtze River basin to South China and Paniin, Liaoning province (Figure 1B) [48]. In summary, the resistance of *C. suppressalis* to chlorantraniliprole has progressively escalated across multiple regions of China. The rapid emergence of resistance highlights the need for continued monitoring and management strategies to mitigate resistance development and ensure the long-term efficacy of diamide insecticides.

## 3. The Resistance Mechanisms of *C. suppressalis*

**Figure 2:**
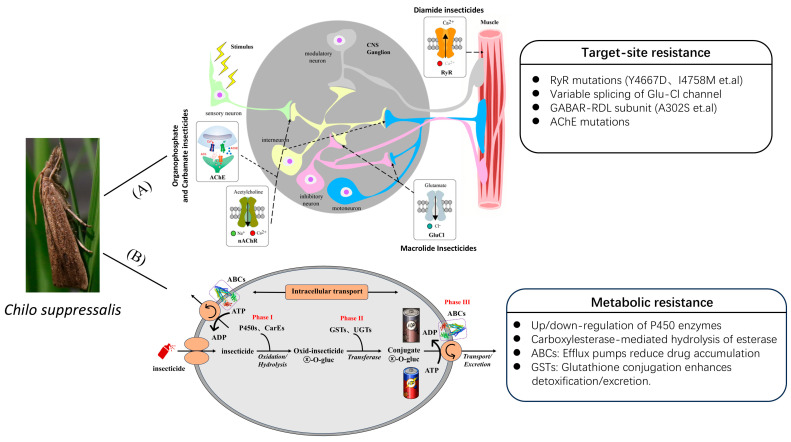
Diagrammatic abstract for the mechanisms of insecticide resistance in *C. suppressalis*. (**A**) Target-site resistance is predominantly driven by mutations in the ryanodine receptor, glutamate-gated chloride channel, GABA receptor, and acetylcholinesterase. These mutations impair insecticide binding by altering the structure or function of the target proteins. (**B**) Metabolic resistance involves three phases: Phase I (oxidative or hydrolytic modifications by cytochrome P450 monooxygenases (P450s) and carboxylesterases (CarEs)), Phase II (conjugation by glutathione S-transferases (GSTs) and UDP-glycosyltransferases (UGTs)), and Phase III (efflux via ATP-binding cassette (ABC) transporters), collectively enhancing detoxification and reducing insecticide accumulation.

The development of resistance in *C. suppressalis* can primarily be attributed to the increasing diversity of insecticides, coupled with their widespread and high-dose application, which has led to corresponding genetic changes within the insect population. Based on the underlying mechanisms of resistance, two primary forms can be identified: target-site resistance and metabolic resistance (Figure 2).

### 3.1. Target-Site Resistance

Target-site resistance is conferred by mutations in the insecticide target receptor or modifications in the expression of the corresponding target protein, which diminishes the binding affinity and thereby confers resistance to the insecticide.

#### 3.1.1. Acetylcholinesterase (AChE)

AChE is a pivotal hydrolytic enzyme in the insect nervous system, responsible for the rapid degradation of the neurotransmitter acetylcholine to terminate cholinergic synaptic transmission [49]. Both organophosphate and carbamate insecticides act by targeting AChE [50]. These insecticides form stable phosphorylated or carbamylated complexes with AChE, thereby inhibiting its catalytic activity, resulting in acetylcholine accumulation in the synaptic cleft, disruption of nerve impulse transmission, and ultimately insect death [51].

In organophosphate-resistant populations of *C. suppressalis*, AChE exhibits markedly reduced sensitivity to compounds such as methamidophos, suggesting that target-site mutations may underlie altered insecticide binding affinity [51]. Subsequent studies identified two AChE-encoding genes in *C. suppressalis*, *ace1* and *ace2*. A point mutation, A314S, in *ace*1 was found to be prevalent in triazophos-resistant populations, with a mutation frequency positively correlated with resistance levels. Structural modeling suggests that this substitution alters the spatial configuration of the catalytic triad serine residue, thereby reducing the enzyme’s affinity for both substrates and inhibitors [52]. In a field population from Taiwan exhibiting extremely high resistance to carbofuran (>1000-fold), five mutations in *ace*1 (E101D, A314S, F402V, R667Q, and H668P) were detected, with substantially higher frequencies in resistant strains compared to susceptible controls (Table 1) [53].

Enhanced AChE activity is widely recognized as a major resistance mechanism against carbamate insecticides [49]. Upon insecticide exposure, increased AChE enzymatic activity often serves as a biomarker of resistance in field populations. Numerous studies have reported a strong positive correlation between AChE activity and resistance levels in various insect pests, including *Aedes albopictus* and *C. suppressalis* [54]. These findings collectively underscore that both target-site mutations and enhanced enzymatic activity of AChE play central roles in mediating resistance to organophosphate and carbamate insecticides in *C. suppressalis*.

#### 3.1.2. Ryanodine Receptor (RyR)

RyR is the primary molecular target of diamide insecticides, and accumulating evidence indicates that point mutations within this receptor constitute a major mechanism of resistance in lepidopteran pests [55]. In *C. suppressalis*, several RyR mutations (G4915E, Y4667D/C, I4758M, and Y4891F) have been shown to be significantly associated with reduced susceptibility to chlorantraniliprole [44,46,56].

Functional validation using CRISPR/Cas9 in *Drosophila melanogaster* and the development of near-isogenic lines in *C. suppressalis* have confirmed that these mutations confer high-level resistance, with the Y4667C/I4758M double mutation conferring significantly higher levels of resistance to both chlorantraniliprole and tetrachlorantraniliprole compared to single-mutation lines, suggesting a possible synergistic effect and highlighting the potential for multiple RyR mutations to collectively drive resistance in field populations [46,47,48,57]. Structural modeling based on the crystallographic analysis of rabbit RyR1 further supports the functional significance of these mutations. And revealed that key residues such as G4915E, I4758M, Y4667C/D, and Y4891F are situated in close proximity to the insecticide-binding pocket. Mutations at these sites are likely to interfere with ligand binding affinity, thereby diminishing insecticidal potency (Table 1) [58].

In addition to structural alterations, post-transcriptional regulation has emerged as a crucial layer in the modulation of RyR expression and resistance phenotypes. In particular, microRNAs (miRNAs) have been implicated in the fine-tuning of target gene expression in response to insecticidal pressure. In a recent study demonstrated that Csu-miR-375 and Csu-miR-11631 were significantly downregulated in chlorantraniliprole-resistant *C. suppressalis* populations, while their predicted target gene, *CsRyR*, was concurrently upregulated. These findings suggest that suppression of miRNA-mediated regulation may contribute to increased RyR expression and enhanced resistance [59].

Collectively, these studies provide compelling molecular evidence that both RyR point mutations and regulatory modulation via miRNAs contribute to the evolution and persistence of diamide resistance in *C. suppressalis.* Understanding these mechanisms is critical for informing resistance monitoring and the development of durable insecticide strategies.

#### 3.1.3. Glutamate-Gated Chloride Channels (GluCls)

Avermectins exert their insecticidal activity primarily by targeting glutamate-gated chloride channels (GluCls), which are widely recognized as their principal molecular targets [60]. In recent years, studies on *C. suppressalis* have increasingly focused on GluCl-mediated mechanisms of resistance, particularly those involving gene expression regulation and alternative splicing.

Accumulating evidence indicates a strong association between *GluCl* gene expression levels and susceptibility to avermectins. Beyond differential expression, alternative splicing of *GluCl* transcripts has been proposed as a key regulatory mechanism modulating insect sensitivity. Although most insect species possess a single *GluCl* gene, alternative splicing and RNA editing can generate multiple functional isoforms, which in turn influence receptor pharmacodynamics. In species such as *D. melanogaster*, *Bombyx mori*, *Laodelphax striatellus*, and *P. xylostella*, splice variants differ primarily in the composition of exons 3 and 9 [61]. Notably, in *P. xylostella*, pharmacological assays revealed that splice variants containing exons 9b and 9c exhibited 2.2-fold and 8.2-fold reduced sensitivity to avermectins, respectively, highlighting the functional significance of isoform composition [62].

In *C. suppressalis*, nine *GluCl* splice variants were identified based on different exon 3 and 9 combinations. Field population analyses demonstrated that the frequencies of exons 3A and 9C were negatively correlated with emamectin benzoate resistance, whereas exons 3B and 9A showed positive associations. Moreover, overall *GluCl* expression levels in resistant field populations were approximately twice those in susceptible strains, suggesting that both overexpression and alternative splicing may cooperatively contribute to resistance. In some isoforms, splice-associated alterations may affect the structural conformation of receptor domains, potentially reducing ligand-binding affinity and thereby diminishing the toxicity of emamectin benzoate. These findings provide a molecular basis for the role of *GluCl* variation in avermectin resistance [63].

In addition to transcriptional and post-transcriptional regulation, point mutations in *GluCl* genes have been implicated in resistance across various pest species. For instance, in resistant populations of *P. xylostella*, three mutations, A309V, G315E, and V263I, were identified, each significantly reducing receptor sensitivity to avermectins by 4.8, 493, and 6.9-fold, respectively, as demonstrated by in vitro electrophysiological assays [64,65,66]. In our laboratory, a D339G mutation was recently detected in field populations of *C. suppressalis* exhibiting avermectin resistance; preliminary data suggest a positive correlation between mutation frequency and resistance level [67].

Collectively, while GluCl-mediated resistance mechanisms in *C. suppressalis* remain under active investigation, current evidence supports the involvement of altered gene expression, splice variant diversity, and potentially resistance-conferring point mutations in the evolution of resistance to avermectin-based insecticides.

### 3.2. Metabolic Resistance

Metabolic resistance refers to the enhanced ability of insects to detoxify insecticides via the upregulation of endogenous detoxification enzymes, thereby accelerating the biotransformation of insecticidal compounds and reducing their toxic efficacy. This resistance mechanism is generally categorized into three sequential phases (Figure 2). In Phase I, insecticides undergo oxidative or hydrolytic modifications primarily catalyzed by cytochrome P450 monooxygenases (P450s), carboxylesterases (CarEs), and flavin-containing monooxygenases. Phase II involves conjugation or substitution reactions mediated by enzymes such as glutathione S-transferases (GSTs) and UDP-glycosyltransferases (UGTs), which increase the solubility of intermediate metabolites. In Phase III, the resulting metabolites are actively transported out of cells via ATP-binding cassette (ABC) transporters, thereby reducing their intracellular accumulation and toxicity [68].

#### 3.2.1. Cytochrome P450 Monooxygenases

Compared to target-site resistance conferred by structural alterations in receptor proteins, metabolic resistance resulting from enhanced detoxification enzyme activity is more frequently reported and exhibits greater prevalence across diverse insect species [69]. In *C. suppressalis*, detoxification enzyme systems have been demonstrated to play a pivotal role in resistance to organophosphate insecticides. For instance, resistance levels to compounds such as methamidophos have been shown to correlate positively with elevated activities of cytochrome P450 monooxygenases (P450s). The use of synergists to inhibit this enzyme significantly restored insecticide susceptibility, confirming their contribution to resistance [51].

In particular, P450 activity was markedly higher in populations showing high-level resistance to triazophos, with a strong positive correlation between enzyme activity and resistance ratio. Inhibition of P450s significantly increased the susceptibility of resistant populations to triazophos. Further molecular analyses revealed that several P450 genes, such as *CYP324A12*, *CYP321F3*, and *CYP9A68*, were significantly upregulated in resistant strains, suggesting that overexpression of these genes underlies metabolic resistance mechanisms [15].

More and more evidence has demonstrated that resistance to diamide insecticides in insects is closely associated with the enhanced activity of multiple detoxification enzymes. In *C. suppressalis*, synergist bioassays using piperonyl butoxide (PBO, a P450 inhibitor) revealed synergistic ratios of 12.4 in field populations resistant to chlorantraniliprole, indicating that elevated activities of microsomal oxidases and esterases contribute significantly to resistance development [70]. Comparative transcriptomic analyses between resistant and susceptible strains identified several upregulated cytochrome P450 genes, including *CYP6CV5*, *CYP9A68*, *CYP321F3*, and *CYP324A12*. Functional validation via RNA interference confirmed that silencing these genes significantly increased the susceptibility of resistant populations to chlorantraniliprole [71]. Subsequent heterologous expression and metabolic assays demonstrated that these four P450s were capable of metabolizing chlorantraniliprole at varying rates [72].

Current evidence suggests that metabolic resistance may play a central role in mediating *C. suppressalis* resistance to diacylhydrazine insecticides. Guo et al. demonstrated that treatment of resistant *C. suppressalis* strains with the cytochrome P450 monooxygenase (P450) inhibitor piperonyl butoxide (PBO) significantly increased their susceptibility to methoxyfenozide, indicating a critical role of P450 expression or enzymatic activity in resistance development. Subsequent transcriptomic analyses identified six P450 genes—*CYP321F3*, *CYP6CV5*, *CYP9A68*, *CYP6AB45*, *CYP324A12*, and *CYP6SN2*—that were highly upregulated in resistant strains. Functional validation through transgenic overexpression in *D. melanogaster* revealed that *CYP321F3* conferred approximately a sevenfold increase in methoxyfenozide resistance, suggesting its key role in metabolic detoxification of benzoylureas in *C. suppressalis* (Table 1) [39].

#### 3.2.2. Carboxylesterases

CarEs mediate resistance development either through the direct hydrolysis of pesticides or by sequestering them, a process in which the insecticide is bound but not efficiently metabolized. CarE-mediated resistance in *C. suppressalis* was first shown for diazinon, an organophosphate insecticide. Biochemical assays revealed that resistant populations displayed significantly elevated activities of CarEs, implicating their involvement in detoxification-mediated resistance [73].

#### 3.2.3. Glutathione S-Transferases

GSTs act on the secondary products produced by other detoxifying enzymes, including P450s and CarEs. Their primary activity is to catalyze the conjugation of the thiol group of glutathione (GSH) to molecules containing an electrophilic center. This reaction enhances water solubility, thereby facilitating their elimination from the insect body [74]. GSTs are reported for their role in insecticide resistance in *C. suppressalis*. In an abamectin-resistant strain of *C. suppressalis*, a positive correlation between the activity of GSTs and resistance levels in field-evolved populations has been reported, indicating that enhanced metabolic enzyme activity may contribute to abamectin resistance [15]. Notably, the use of GST inhibitors significantly increased the toxicity of abamectin against *C. suppressalis*, further supporting the role of GST-mediated detoxification in resistance development [75].

#### 3.2.4. UDP-Glycosyltransferases

UGTs can conjugate lipophilic endogenous and xenobiotic substrates into more water-soluble glycosylated compounds and thus may drive the evolution of insecticide resistance [76]. In resistant *C. suppressalis* strains, the expression levels of *CsUGT40AL11* and *CsUGT33AG3* were found to be 12- and 5-fold higher, respectively, than those in susceptible strains. RNAi-mediated knockdown of these genes significantly increased sensitivity to chlorantraniliprole [77]. Similarly, overexpression of *UGT2B17* was implicated in chlorantraniliprole resistance in *Plutella xylostella*, suggesting that UGTs may participate in a conserved metabolic resistance mechanism across lepidopteran pests [78].

#### 3.2.5. ATP-Binding Cassette

ABC transporters, which constitute the third phase of detoxification, have also been implicated in resistance to multiple insecticides. Biochemical evidence from synergist assays confirms their functional role in resistance modulation, a finding supported by transcriptomic analyses that consistently identify the upregulation of specific ABC transporter genes (*CsABCA3*, *CsABCC1*, *CsABCC8*, and *CsABCH1*) in resistant populations. Functional validation in insect cell lines demonstrates that the overexpression of these genes, particularly Cs*ABCC8* and C*sABCH1*, directly reduces cellular toxicity to multiple insecticides, including chlorantraniliprole, cyantraniliprole, and abamectin [77,79,80,81]. The induction of these transporters upon insecticide exposure and their elevated expression in field-resistant strains further underscores their adaptive role. Collectively, these findings establish that the overexpression of specific ABC transporters, leading to increased efflux of toxic compounds, is a primary mechanism of multi-insecticide resistance in this pest.

#### 3.2.6. Flavin-Containing Monooxygenases

In addition to P450s, flavin-containing monooxygenases (FMOs), a class of non-P450 oxidative enzymes involved in xenobiotic metabolism, have been linked to metabolic resistance. In chlorantraniliprole-resistant *C. suppressalis* populations, FMO3B and FMO3C were found to be highly upregulated. Functional assays in transgenic Drosophila demonstrated that these FMOs conferred resistance to chlorantraniliprole, and RNAi knockdown of either gene significantly increased insecticide susceptibility, suggesting a detoxification role for FMOs in mediating resistance [37].

Collectively, enhanced activities of detoxification enzymes-including P450 monooxygenases, esterases, UGTs, ABC transporters, and FMOs-represent key molecular mechanisms underpinning diamide resistance in *C. suppressalis*. These findings provide a mechanistic foundation for resistance monitoring and the development of targeted management strategies.

**Table 1:** Episodes of field resistance to multiple insecticides in *C. suppressalis* in China.

Insecticides	Resistance Ratios	Target-Site Resistance	Metabolic Resistance	Functional Validation	References
Triazophos	68.7	_	*CYP324A12*, *CYP321F3* and *CYP9A68*	Synergism experiment and qRT-PCR	[15]
Carbofuran	>1000	E101D A314S F402V R667Q H668P	_	Enzyme kinetics and inhibition assays	[52,53]
Methoxyfenozide	>100	_	*CYP321F3*	Synergism experiment, qRT-PCR, and transgenic expression in *Drosophila melanogaster*	[39]
Chlorantraniliprole	82.37	_	*CYP6CV5*, *CYP9A68*, *CYP321F3* and *CYP324A12*	RNAi	[71]
Chlorantraniliprole	44.32	_	*UGT40AL11* and *UGT33AG3*	RNAi	[77]
Chlorantraniliprole	77.6	G4910E		Bioassay and sequencing of *Cs*RyR	[44]
Fubendiamide	42.6	G4910E		Bioassay and sequencing of *Cs*RyR	[44]
Chlorantraniliprole	249.6	Y4667D Y4667C I4758M		Bioassay and sequencing of *Cs*RyR	[56]
Chlorantraniliprole	102.9–536.8	Y4667D/C I4758M G4915E Y4891F		Bioassay, sequencing of *Cs*RyR and CRISPR/Cas9 genome-modified *Drosophila melanogaster*	[46]
Chlorantraniliprole	109.6–2087.5	I4758M and Y4667C		Bioassay, sequencing of *Cs*RyR and CRISPR/Cas9 genome-modified *Drosophila melanogaster*	[47]
Tetraniliprole	27.7–806.8	Y4667D/C I4758M G4915E Y4891F		Bioassay, sequencing of *Cs*RyR and CRISPR/Cas9 genome-modified *Drosophila melanogaster*	[57]
Chlorantraniliprole	111.6–2706.4	Y4667D		Bioassay, introgression of the *Cs*RyR 4667D allele into the susceptible strain and molecular docking	[48]

## 4. *C. suppressalis* Resistance Management

Due to the prolonged and frequent application of chemical insecticides, resistance has been a significant concern, and relying on chemical control only cannot achieve good efficacy against *C. suppressalis*. Resistance management for *C. suppressalis* encompasses a range of strategies, including agronomic, biological, physical, and chemical control methods, as well as integrated pest management (IPM) approaches that combine these tactics for enhanced effectiveness. To delay the development of resistance and preserve the efficacy of available insecticides, it is essential to develop scientific and rational resistance management strategies. This involves strengthening resistance monitoring systems and platforms and providing special guidance for pesticide application in the local area because the resistance of *C. suppressalis* shows strong regional characteristics. Currently, some successful strategies are employed in the management of *C. suppressalis* resistance.

The application of seed coating or seed dressing technologies has proven effective in managing early-season insect pests in rice cultivation. Seed coating involves encapsulating the seed surface with a formulation containing insecticides, fertilizers, and plant growth regulators, while seed dressing refers to the direct mixing of insecticides with seeds to facilitate surface adherence. Recent studies have shown that seed treatment with chlorantraniliprole provides over 90 days of effective control against major rice pests, including *C. suppressalis*, *Cnaphalocrocis medinalis*, and *Sesamia inferens*. Moreover, chlorantraniliprole seed treatment significantly elevated endogenous jasmonic acid (JA) levels in rice plants, enhancing plant defense responses and increasing larval mortality of *C. suppressalis*, thereby synergistically improving the insecticidal efficacy of the compound [82].

The “insecticide-treated seedling” technique involves treating rice seedlings with appropriate insecticides prior to transplanting, enabling the seedlings to retain residual pesticide activity upon field establishment. This approach provides early-season protection by preventing or mitigating pest outbreaks during the initial growth stages after transplantation [83]. For instance, application of the novel, long-residual diamide insecticide tetrachlorantraniliprole to seedlings demonstrated over 85% corrected efficacy against *C. suppressalis* infestations in early-season rice fields of Hangzhou (Zhejiang) and Hengyang (Hunan) at 35 days post-transplantation. This treatment significantly reduced post-transplantation damage by *C. suppressalis*, ultimately enhancing seedling survival and plant vigor [84].

The rational use of sex pheromones, applied at optimal timings, can significantly enhance the effectiveness of pest control measures. In the case of *C. suppressalis*, sex pheromones can be used to trap and kill adult moths, thereby reducing the pest population density in the field. During peak emergence periods, the strategic placement of these traps can effectively reduce mating opportunities, leading to a decrease in the number of larvae in subsequent generations. The judicious use of sex pheromones not only diminishes the reliance on chemical insecticides but also reduces their impact on beneficial insect populations, thereby supporting the maintenance of ecological balance [85,86,87]. Recent studies have highlighted the efficacy of using active high-dose aerosol pheromone dispensers for mating disruption to control *C. suppressalis* in rice fields. This approach has proven to be highly effective in controlling *C. suppressalis* populations and mitigating crop damage, with a larval reduction rate of 95.1% and a significant decrease in rice damage [88,89,90].

The rational use of novel insecticides with distinct modes of action is a critical strategy for managing pesticide resistance in pest populations. The identification and development of new active ingredients to control resistant pest species, alongside a comprehensive understanding of their mechanisms of action, remain central objectives in the global insecticide research and development landscape. Such efforts have led to a gradual yet steady increase in the diversity of insecticide target sites over the past seven decades, expanding from three target sites in the 1950s to 22 distinct target sites by 2018 [91]. These new insecticides typically feature high efficacy, low toxicity, and environmental friendliness, offering promising solutions for the management of various resistant pest populations. Insecticides targeting novel sites of action, such as cyproflanilide [92,93], broflanilide [94,95,96], and dimpropyridaz [97], have demonstrated the ability to circumvent cross-resistance with traditional insecticides, showing effective control over resistant populations of pests. It is essential, however, to fully understand the mode of action, application range, and usage guidelines of these new insecticides to ensure their effective and sustainable use.

The application of natural enemies, specifically parasitic wasps, for the biocontrol of rice pests, including *C. suppressalis*, plays a pivotal role in integrated pest management. Parasitic wasps lay their eggs within the larvae or eggs of *C. suppressalis*, and the developing wasp larvae eventually kill the host. Numerous parasitic wasps have been identified as effective biocontrol agents against *C. suppressalis*, including *Trichogramma japonicum*, *Trichogramma chilonis*, *Trichogramma dendrolimi*, *Trichogramma ostriniae*, and *Cotesia chilonis* [98]. Laboratory cage trials have demonstrated that *T. japonicum* and *T. chilonis* exhibit high parasitism rates on *C. suppressalis* egg masses. Furthermore, field experiments involving the release of these two species have also shown promising control effects. The use of parasitic wasps offers significant advantages in terms of environmental sustainability, as it does not contribute to environmental pollution and reduces the reliance on chemical insecticides. However, the effectiveness of parasitic wasps in controlling *C. suppressalis* is influenced by various factors, including field conditions and host density. Therefore, further research is required to optimize release techniques and environmental conditions to maximize their efficacy in pest control [99,100,101].

The application of entomopathogenic fungi in pest management, particularly for *C. suppressalis*, holds significant potential. Entomopathogenic fungi are a group of fungi that infect and kill insects, offering a promising alternative for pest control. Studies have shown that various entomopathogenic fungi, such as *Beauveria bassiana* and *Metarhizium anisopliae*, exhibit pathogenicity against *C. suppressalis* [102,103]. These fungi penetrate the insect’s exoskeleton to invade the host’s body, where they grow and reproduce, ultimately causing the death of the insect. For instance, infection with *B. bassiana* leads to the production of toxins inside the *C. suppressalis* body, disrupting its physiological functions. Entomopathogenic fungi offer several advantages, including a broad host range and environmental compatibility. Furthermore, their insecticidal efficacy can be enhanced through biotechnological methods, such as genetic engineering. However, their application faces certain challenges. Environmental factors, such as temperature and humidity, significantly influence their activity, which must be carefully considered when applying these fungi in field conditions. Additionally, entomopathogenic fungi are safe for parasitoid wasps, and when these fungi’s conidia are carried by the parasitoid wasps, they can enhance the biological control of *C. suppressalis* [104,105].

The use of *Vetiveria zizanioides* (vetiver grass) can be an important component in the integrated pest management of *C. suppressalis*. Vetiver grass exhibits a certain attractant effect on this pest, making it a valuable tool for pest control. By planting vetiver grass around the perimeter of rice fields or within the fields themselves, *C. suppressalis* adults are drawn to lay their eggs on the vetiver grass, thereby reducing oviposition on rice plants and consequently mitigating the damage caused by this pest [106,107,108]. Studies have shown that the volatile compounds released by vetiver grass attract female *C. suppressalis* moths to lay their eggs; however, the larvae that hatch from these eggs are unable to complete their life cycle on the vetiver grass [109,110]. Moreover, vetiver grass provides a habitat for the natural enemies of *C. suppressalis*, supporting their refuge and reproductive needs, which enhances their pest control capabilities. For example, in some regions, the cultivation of vetiver grass along field embankments has effectively reduced the population of *C. suppressalis* [87,111]. Additionally, combining the strategic placement of vetiver grass with other control measures, such as the installation of sex pheromone traps, has been shown to significantly reduce the pest damage in rice fields by 70–87%. This integrated approach improves the overall efficacy of *C. suppressalis* control while minimizing the use of chemical pesticides, contributing to sustainable agricultural practices [87].

## 5. Conclusions and Prospects

This paper reviews the insecticide resistance status and resistance mechanisms of *C. suppressalis*. At present, the application of insecticides is the main approach to control *C. suppressalis* because of their efficacy and convenience. However, the widespread insecticide resistance poses a serious challenge to farmers and scientists.

*C. suppressalis* has evolved resistance to insecticides from most chemical classes, including avermectins, organophosphates, macrolides, diamides, and diacylhydrazines. Among the commonly used insecticides in the field, the resistance of *C*. *suppressalis* to diamide insecticide is more serious than that of other types of insecticides. The mechanisms of resistance in *C*. *suppressalis* are similar to those that have been described from many other pest species, and they are generally classified as point mutations in the target site, or metabolic, involving esterase-, glutathione S transferase-, P450 monooxygenase-, UDP-glycosyltransferases-, flavin-containing monooxygenases-, or ATP-binding cassette transporters-based detoxification. Applying advanced methods to analyze molecular and gene sequence data obtained from resistant and susceptible field-collected *C. suppressalis* populations has enabled a better understanding of the resistance mechanisms in this pest.

There are several components of Insecticide Resistance Management (IRM) programs applicable to managing the resistance in *C*. *suppressalis*, namely chemical control with rotation of MoAs, insecticide-treated seedlings, and nonchemical control methods, such as biological control, rice plant resistance, and physical/mechanical methods. It is hoped that the integration of these methods will contribute to improving the management of this pest, thereby helping to ensure rice’s sustainable yields in the future.

## Data Availability

No new data were created or analyzed in this study.
